# Australia's first fossil marsupial mole (Notoryctemorphia) resolves controversies about their evolution and palaeoenvironmental origins

**DOI:** 10.1098/rspb.2010.1943

**Published:** 2010-11-03

**Authors:** Michael Archer, Robin Beck, Miranda Gott, Suzanne Hand, Henk Godthelp, Karen Black

**Affiliations:** 1 School of Biological, Earth and Environmental Sciences, University of New South Wales, Sydney 2052, Australia; 2 Department of Mammalogy, American Museum of Natural History, Central Park West at 79th Street, New York, NY 10024, USA

**Keywords:** Australian marsupial moles, Riversleigh Miocene fossil, evolutionary convergence, zalambdodonty, fossorial adaptations, rainforest palaeohabitat

## Abstract

Fossils of a marsupial mole (Marsupialia, Notoryctemorphia, Notoryctidae) are described from early Miocene deposits in the Riversleigh World Heritage Area, northwestern Queensland, Australia. These represent the first unequivocal fossil record of the order Notoryctemorphia, the two living species of which are among the world's most specialized and bizarre mammals, but which are also convergent on certain fossorial placental mammals (most notably chrysochlorid golden moles). The fossil remains are genuinely ‘transitional', documenting an intermediate stage in the acquisition of a number of specializations and showing that one of these—the dental morphology known as zalambdodonty—was acquired via a different evolutionary pathway than in placentals. They, thus, document a clear case of evolutionary convergence (rather than parallelism) between only distantly related and geographically isolated mammalian lineages—marsupial moles on the island continent of Australia and placental moles on most other, at least intermittently connected continents. In contrast to earlier presumptions about a relationship between the highly specialized body form of the blind, earless, burrowing marsupial moles and desert habitats, it is now clear that archaic burrowing marsupial moles were adapted to and probably originated in wet forest palaeoenvironments, preadapting them to movement through drier soils in the xeric environments of Australia that developed during the Neogene.

## 1. Introduction

Notoryctemorphia, the marsupial moles, is the least diverse but most extraordinarily distinct of the four orders of living Australian marsupials. It currently comprises one family (Notoryctidae), one genus (*Notoryctes*) and two species (*Notoryctes typhlops, Notoryctes caurinus*). Until the discovery of the extinct taxon described here, no fossil notoryctemorphians were known [1]. In part because of this lack of a fossil record, the origins and relationships of the group have long been the subject of speculation and debate, with some authors even questioning their marsupial status [2–4]. Most recent studies have supported a close relationship between Notoryctemorphia and Dasyuromorphia (Australian carnivorous marsupials [5]), Peramelemorphia (bandicoots and bilbies [6,7]) or both [8–13].

**Figure 1.**
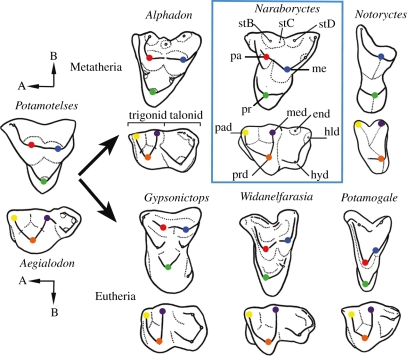
Cusp evolution from tribosphenic to zalambdodont patterns in representative metatherians (top row) and eutherians (bottom row), and dental terminology used in text. *Naraboryctes philcreaseri* n. gen. and sp. indicates that in zalambdodont metatherians the paracone (red) is suppressed; in eutherians it is the metacone (blue) that is reduced. Upper and lower molars redrawn from: [63] (*Gypsonictops*); [64] (*Potamotelses*, *Aegialodon*, *Alphadon*); [65] (*Widanelfarasia* lower); [66] (*Potamogale*); [67] (*Widanelfarasia* upper). Abbreviations: A, anterior; B, buccal; end, entoconid; hld, hypoconulid; hyd, hypoconid; me, metacone; med, metaconid; pa, paracone; pad, paraconid; pr, protocone; prd, protoconid; stB, stylar cusp B; stC, stylar cusp C; stD, stylar cusp D.

**Figure 2.**
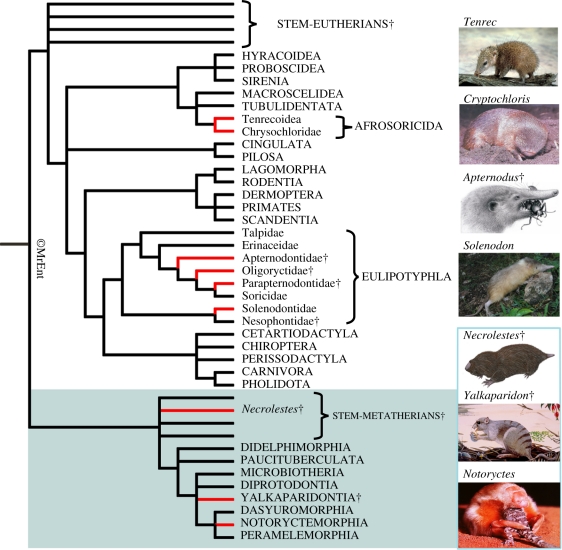
Composite phylogeny of therian mammals illustrating the occurrence of dental zalambdodonty within Theria. The topology is based on recent studies including [11–13,15,19,20,68–72]. Taxa that include at least one fully zalambdodont member are indicated by red branches. Extinct taxa are indicated by a dagger. Images (from top to bottom) are: the tenrecoid *Tenrec ecaudatus* (J. F. Eisenberg; AMS Image Library); an unidentified chrysochlorid *Cryptochloris* sp. (Wikimedia Commons image; Killer18); the apternodontid *Apternodus baladontus* (frontispiece [17]: p. 2); the solenodontid *Solenodon paradoxus* (J. Nuñez-Miño; www.thelastsurvivors.org); the necrolestid *Necrolestes patagonensis* (N. P. Archer); the yalkaparidontian *Yalkaparidon coheni* (D. Dunphy); the notoryctid *Notoryctes typhlops* (M. Gillam). The phylogeny was created using the phylogenetic drawing tool MrEnt 2.0 [73].

Among living marsupials, *Notoryctes* is unique in exhibiting a distinctive molar morphology termed zalambdodonty [14,15]. Zalambdodonty is characterized by upper molars having a single, central cusp homologous with either the paracone or the metacone [15,16]. Two crests extend buccally from this cusp, resulting in a v-shaped crown (figure 1). In addition, the protocones (upper molars) and talonids (lower) are usually reduced or lost. Besides *Notoryctes*, several living and fossil therian groups have zalambdodont molars (figure 2); these include extant solenodontids, chrysochlorids and tenrecids (all placentals) and fossil apternodontids (which also appear to be placentals [17]), *Yalkaparidon* (probable australidelphian marsupial [11,18]) and *Necrolestes* (probable metatherian [19,20]). Without a fossil record to reveal intermediate conditions, there has been uncertainty about which of the upper molar cusps *Notoryctes* has lost in comparison to other zalambdodont taxa. The question is interesting in terms of evolutionary process: do mammals evolving zalambdodonty (or any other comparably specialized dental morphology) become increasingly ‘channelled’ by an underlying morphological/genetic developmental constraint as they move down this pathway, or can these highly specialized patterns be achieved in completely different ways? That is, do they arise by parallelism (independent acquisition via the same evolutionary pathway) or convergence (independent acquisition via a different pathway)? While most placental mammals have achieved zalambdodonty via hypertrophy of the paracone and suppression of the metacone [15–17], until now there has been no hard (fossil) evidence for how this pattern has been achieved in marsupials.

Modern notoryctids are confined to deserts in Australia and exhibit numerous anatomical specializations that appear exceptionally well suited to burrowing through desert sands. These include a conical skull, extreme modifications of the axial and appendicular skeleton (in particular, enormous enlargement of the muscle attachment sites on the fore- and hindlimbs) and soft tissue features such as a ‘nasal shield’, a lack of eyes and external ears, and a tubular body shape [21]. As a result, it has long been assumed that notoryctids evolved these specializations in a desert environment. This led to speculation (e.g. [22]) that a desert palaeoenvironment must have existed somewhere in Australia throughout much of the Cenozoic to allow for the evolution of the highly modified, autapomorphic notoryctid body form, despite no direct evidence for sandy deserts in Australia prior to the Pleistocene [23,24]. Notoryctids have been used as text-book examples of convergence between themselves and the phylogenetically unrelated but morphologically very similar placental golden moles (chrysochlorids; e.g. [25,26]). As well as zalambdodont molars, *Notoryctes* and chrysochlorids share similar fossorial specializations of the skeleton [19,26] and closely resemble each other in terms of external appearance (figure 2). Like *Notoryctes*, some (but not all) chrysochlorids occupy sandy desert environments [27]. This fact contributed to earlier presumptions (e.g. [22]) that the similar overall morphology of both groups is the result of long-term adaptation to desert environments. Until now it has not been possible to test this presumption on the basis of palaeoenvironmental data.

Higher level systematic nomenclature used in this paper follows [28]. Dental terminology follows [18,29] for crown morphology (figure 1) and [30] for molar number. Case denotes upper (e.g. M2) and lower (e.g. m2) teeth.

## 2. Systematic palaeontology

Mammalia Linnaeus, 1758

Marsupialia Illiger, 1811

Notoryctemorphia Aplin & Archer, 1987

Notoryctidae Ogilby, 1892

*Naraboryctes philcreaseri* new genus and species.

### (a) Etymology

From *naraba* ‘to drink’ (Garrawa and Waanyi languages of northwestern Queensland; [31]), in reference to its rainforest palaeohabitat, and *oryctes* meaning ‘digger’ (Greek), in reference to its fossorial specializations and close relationship to *Notoryctes*. The species name honours Phil Creaser for many contributions to Riversleigh and other palaeontological research at the University of New South Wales including establishment of the Coalition for Research into Australian Terrestrial Ecosystems (CREATE) Fund.

### (b) Holotype

**Figure 3.**
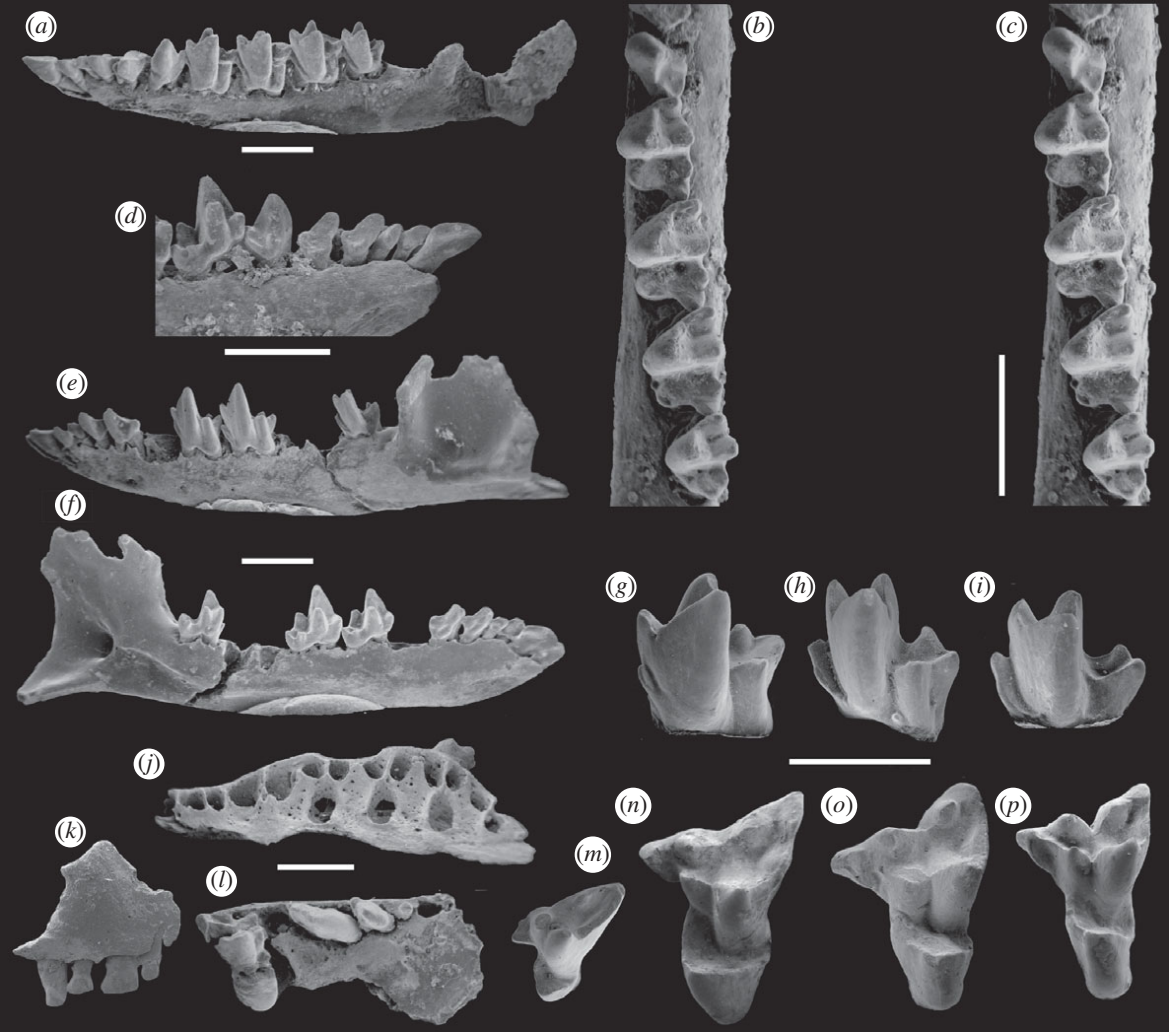
*Naraboryctes philcreaseri* new genus and species Riversleigh World Heritage Area, northwestern Queensland, Australia; early Miocene. (*a*–*d*) QM F23717, holotype, left dentary with i2-m4; (*a*) buccal view; (*b*,*c*) stereopair occlusal view; (*d*) lingual view of anterior dentition. (*e*,*f*) QM F23719, left dentary with i1-p2 m1-2 m4; (*e*) buccal view; (*f*) lingual view. (*g*) QM F23718, left m1. (*h*) QM F51329, left m3. (*i*) QM F51330, right m4 (reversed image). (*j*) QM F54502, left edentulous maxilla. (*k*) QM F51322, paratype, left premaxilla with I1-4. (*l*) QM F23716, paratype, partial right maxilla with P1-3 M1. (*m*) QM F51323, right dP3 (reversed image). (*n*) QM F51324, left M1. (*o*) QM F51325, right M2 (reversed image). (*p*) QM F51327, left M3 (scale bar, 2 mm).

QM F23717 (Queensland Museum) from Upper Site, left dentary with i1-3 (i1 root only) c1 p1-3 m1-4, missing mandibular condyle and medially inflected portion of angular process (figure 3*a*–*d*).

### (c) Paratypes and referred specimens

**Figure 4.**
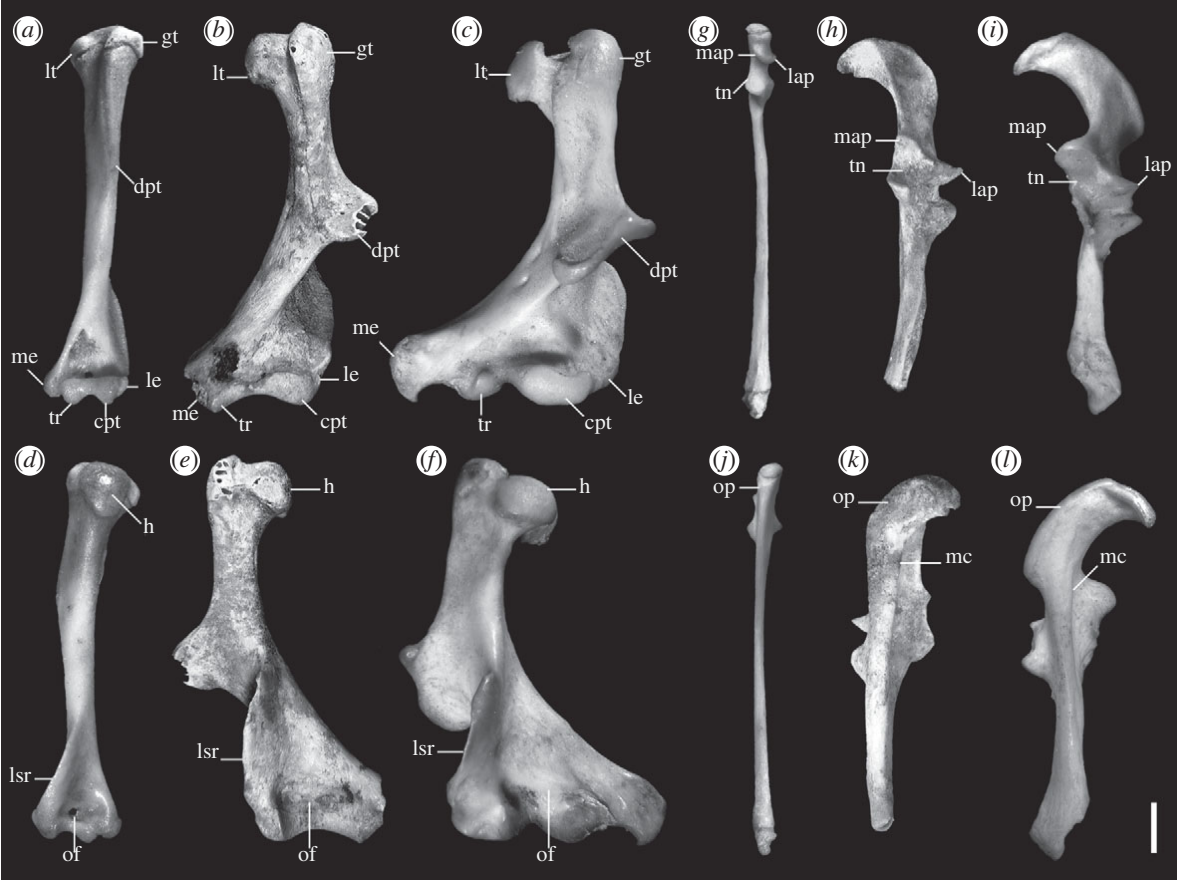
Comparison of humeri and ulnae of *Naraboryctes philcreaseri* gen. et sp. nov., *Notoryctes typhlops* (SAM637) and *Antechinus stuartii* (UNSWZ465). (*a*–*c*) left humerus, anterior view; (*a*) *A. stuartii*; (*b*) *N. philcreaseri* (QM F54559); (*c*) *N. typhlops*. (*d*–*f*) left humerus, posterior view; (*d*) *A. stuartii*; (*e*) *N. philcreaseri* (QM F54559); (*f*) *N. typhlops*. (*g*–*i*) left ulna, anterior view; (*g*) *A. stuartii*; (*h*) *N. philcreaseri* (QM F54560); (*i*) *N. typhlops*. (*j*–*l*) left ulna, posterior view; (*j*) *A. stuartii*; (*k*) *N. philcreaseri* (QM F54560); (*l*) *N. typhlops* (scale bar, 2 mm). Mirror image of right humerus and ulna of *A. stuartii* and *N. typhlops* used for comparison. Abbreviations: cpt, capitulum; dpt, delto-pectoral tuberosity; gt, greater tuberosity; h, head; lap, lateral anconeal process; le, lateral epicondyle; lsr, lateral supracondylar ridge; lt, lesser tuberosity; map, medial anconeal process; mc, medial crest; me, medial epicondyle; of, olecranon fossa; op, olecranon process; tn, trochlear notch; tr, trochlea.

Paratypes that are also topotypes: QM F51322, partial left premaxilla with I1-4 (figure 3*k*); QM F23716, partial right maxilla with P1-3 M1 (figure 3*l*). Referred specimens: from Upper Site, QM F54502 (edentulous maxilla; figure 3*j*); QM F51323 (dP3; figure 3*m*), QM F51324 (LM1; figure 3*n*), QM F51325 (RM2; figure 3*o*), QM F51327 (LM3; figure 3*p*), QM F23718 (Lm1; figure 3*g*), QM F51329 (Lm3; figure 3*h*), QM F51330 (Rm4; figure 3*i*), QM F54559 (left humerus; figure 4*b*,*e*), QM F54560 (left ulna; figure 4*h*,*k*); from Wayne's Wok Site, QM F23719 (left dentary; figure 3*e*–*f*), QM F51328 (left dentary).

### (d) Locality and horizon

The Type Locality is Upper Site, Godthelp Hill, Riversleigh World Heritage Area, Boodjamulla National Park, northwestern Queensland (19°0′42″ S, 138°40′24″ E). Wayne's Wok Site is on Hal's Hill and 250 m from Upper Site. Both have been interpreted as Faunal Zone B sites [32–35] and as such early Miocene in age based on biocorrelation and preliminary radiometric dating (U/Pb) of encasing limestone. All Riversleigh specimens have been recovered by acetic acid-processing of limestone.

### (e) Diagnosis and description

The only known species of *Naraboryctes* differs from *Notoryctes* spp. in the following combination of features: retention of small paracone; postmetacrista much longer than premetacrista; medial cusp on postmetacrista variably present; well-developed three-cusped talonid which is slightly smaller than trigonid on m2-4; anterior cingulid present; complete adult dental formula I1-5/1-3, C1/1, P1-3/1-3, M1-4/1-4; proportionately larger coronoid process of dentary; medial epicondyle of humerus less enlarged; capitulum and trochlea continuous; supracondyloid foramen present; elongated olecranon process of ulna less hooked; flat (rather than concave) medial anconeal process of ulna; anconeal sides of capitular notch and trochlear notch of ulna continuous (rather than separated by a ridge).

Rediagnosis of family Notoryctidae: small-bodied (<100 g) marsupials; complete adult dental formula I1-5/1-3, C1/1, P1-3/1-3, M1-4/1-4; upper molars sub- or fully zalambdodont with paracone much smaller than metacone or missing altogether but with large protocone; lower molars with reduced or absent talonids; postcranium with fossorial adaptations. A detailed description of the dentition, cranial and postcranial elements of this new taxon is provided in the electronic supplementary material, appendix S1.

## 3. Palaeoenvironment

Previous authors [33,36–40] have concluded, on the basis of taxic representation, community structure, species morphology and geology that the early Miocene palaeocommunities of Riversleigh represent closed forest environments. Reasons include representation in these assemblages of taxa all living species of which exist only in rainforests (e.g. species of *Menura*, *Orthonyx, Hypsiprymnodon*, dactylopsiline petaurids and phalangerin phalangerids; e.g. [41–43]). Among chiropterans, the very diverse and abundant hipposiderids as well as mystacinids present at Riversleigh during the early Miocene are typical of modern closed forest environments [44,45]. Riversleigh frog assemblages of this age resemble those that today occupy permanently wet rather than dry or even seasonally dry forest environments (M. Tyler 2009, personal communication). Marsupial and bat diversity in Riversleigh assemblages is by modern Australian standards extraordinarily high. Diversity of these groups in Australasia today is highest in rainforest environments such as those in mid-montane New Guinea, where complex guilds based on, for example, size and feeding strategies enable many species to coexist.

## 4. Discussion

We refer *Naraboryctes philcreaseri* to Notoryctemorphia because of the extreme reduction of the paracone, anteroposterior compression of its upper molars and reduction of the talonid on its lower molars. Collectively, these apomorphies represent an incipiently zalambdodont morphology that anticipates the fully zalambdodont condition in species of *Notoryctes*. This familial attribution is further supported by cranial and isolated postcranial specimens referable to *N. philcreaseri* in addition to the premaxilla, humerus and ulna noted above [7,46]. These exhibit numerous adaptations for fossoriality very similar to but less well developed than those seen in species of *Notoryctes* [7,46]. The humerus (figure 4) exhibits enlargement of many sites for muscle attachment, in particular expansion of the medial epicondyle for enlarged flexor muscles that facilitate strong flexion in the wrist during digging by fossorial mammals. As in both species of *Notoryctes*, the delto-pectoral ridge is also markedly displaced distally, resulting in increased mechanical advantage of the musculature, and the enlarged lateral supracondylar ridge provides for increased musculature in the lower arm. Massively expanded articular surfaces help strengthen the joints during digging by spreading high mechanical forces over a greater surface area [7]. The hypertrophied olecranon process of the ulna, which enables powerful extension of the forelimb during digging, while decidedly hypertrophied and notoryctid-like in *N. philcreaseri* (figure 4) and hence a synapomorphy for this family, is not quite as large nor as strongly curved medially as it is in species of *Notoryctes*. Hypertrophy of the olecranon process is present in many fossorial mammals [7,19,26] but is unique to notoryctids within Marsupialia. Preliminary phylogenetic analyses by one of us (R.M.B.D.) indicate that *Naraboryctes* and *Notoryctes* form a clade.

*Naraboryctes philcreaseri* is dentally more plesiomorphic than either species of *Notoryctes*, notably in its apparent retention of five upper incisors, three upper and lower premolars, distinct paracone on the upper molars and three-cusped talonid on the lower molars. As noted above, the dental formula of *N. philcreaseri* appears to be I1-5 (or possibly I1-4; see the electronic supplementary material)/1-3; C1/1; P1-3/1-3; M1-4/1-4. This is the same as the plesiomorphic dental formula for Australian marsupials and, except for the loss of one lower incisor, the same as the most plesiomorphic dental formula known for any marsupial. All known Australian marsupials have lost i4 and all except some peramelemorphians and probably *N. philcreaseri* have lost I5. The dental formula for species of *Notoryctes* is controversial because of considerable polymorphism in tooth number, both between specimens and within the same specimen. While Archer [22] stated that the *maximum* dental formula of *N. typhlops* is I1-4/1-3 C1/1 P1-3/1-3 M1-4/1-4, loss of an upper incisor and at least one upper and one lower premolar is not uncommon. Thomas [47] gave the dental formula of *N. caurinus* as I1-3/1-2; C1/1; P1-2/1-2; M1-4/1-4. The tiny size of P1 in *N. philcreaseri* compared with P2-3 suggests that it is P1 rather than P3 that is lost in *N. caurinus* and some specimens of *N. typhlops*. An apparent autapomorphy of *N. philcreaseri* is the presence of a cuspule near the middle of the postmetacrista, although it is possible that this cuspule has been secondarily lost in species of *Notoryctes*.

The dental material of *N. philcreaseri* unequivocally demonstrates that zalambdodonty evolved in notoryctemorphians from a tribosphenic precursor by loss of the paracone (figure 1). Asher *et al*. [19] recently hypothesized that zalambdodonty in the probable metatherian *Necrolestes* was also acquired by suppression of the paracone. This hypothesis was based on occlusal relations in a fully zalambdodont taxon rather than discovery of an annectent taxon of the kind we describe here. Conversely, although the molars of the Eocene ?metatherian *Kiruwamaq chisu* [48] and some living dasyurids have greatly reduced paracones [49], they are not fully zalambdodont. The fossils of *N. philcreaseri* described here are therefore, to our knowledge, the first direct evidence that full zalambdodonty can be achieved by loss of the paracone rather than the metacone (the cusp lost by zalambdodont placentals). This demonstrates that highly specialized morphological patterns, such as zalambdodonty, can be achieved in very different ways in different mammalian clades and can result from convergent rather than parallel evolution (figure 1).

It is not clear exactly why notoryctemorphians evolved zalambdodonty by suppressing the paracone, whereas zalambdodont placentals (e.g. apternodontids, solenodontids, chrysochlorids, tenrecids) have suppressed the metacone. However, perhaps the simplest explanation is that the metacone is typically larger than the paracone in marsupials and other metatherians, whereas the reverse is usually true for placentals and other eutherians [50], and it is the smaller of the two cusps that is lost in zalambdodont forms. Within placentals, bats are unusual in that the metacone is usually larger than the paracone; it is therefore noteworthy that the bat *Harpiocephalus* has evolved an incipiently zalambdodont morphology by reduction of the paracone rather than the metacone [15].

The functional significance of zalambdodonty and hence the probable feeding ecology of *N. philcreaseri* remain unclear. However, based on the mechanical properties of food items (see [51]), Beck [52] suggested that zalambdodonty may represent a specialization for feeding on soft-bodied invertebrates such as worms or insect larvae. A tribosphenic dentition with a fully functional protocone–talonid complex, by contrast, may be better adapted for feeding on harder food items including adult insects. The natural diet of both species of *Notoryctes* is poorly known [53], although captive specimens appeared to prefer larvae over adult insects [54]. Within placentals, the tenrecid *Hemicentetes*, which shows extreme zalambdodonty ([55], fig. 41D), feeds almost exclusively on earthworms [56] whereas *Potamogale*, which feeds predominantly on hard-shelled crustaceans [27], retains a small metacone as well as a large protocone and relatively well-developed talonids ([15,55], fig. 41A). The presence of a small paracone and a functional talonid in *N. philcreaseri* may therefore indicate that its diet included a greater proportion of harder food items (such as hard-bodied invertebrates) than those of species of *Notoryctes*. Insect larvae and worms are common below ground; if zalambdodonty is indeed an adaptation for feeding on soft-bodied invertebrates, this may explain why many zalambdodont mammals are fossorial (species of *Notoryctes*, *Necrolestes*, chrysochlorids and the tenrecid *Orizoryctes*) or semi-fossorial (species of *Solenodon*, *Hemicentetes* and possibly apternodontids [57]). The coronoid process of the mandible of *N. philcreaseri* is higher than that of either species of *Notoryctes*, which suggests the presence of more powerfully developed temporalis musculature. The coronoid process is very low in the vermivorous species of *Hemicentetes* compared with tenrecids which feed on harder-bodied invertebrates [58]. In bats the coronoid process is smaller in those species that feed on softer-bodied prey such as moths (e.g. [59,60]). The morphology of the coronoid process may therefore be further evidence that *N. philcreaseri* fed on a wider range of food items including some harder prey than do species of *Notoryctes*.

Collectively, the dentition and mandible of *N. philcreaseri* appear to be less-specialized for feeding on soft-bodied invertebrates and hence they may have spent relatively less time feeding underground. However, because powerful digging in this species, as indicated by the hypertrophied, notoryctid-like olecranon process, is combined with incipient zalambdodonty, burrowing in notoryctids probably preceded evolution of fully developed zalambdodonty as well as a concomitant shift in preference for softer subterranean foods.

Recent dated molecular phylogenies suggest that Notoryctemorphia originated in either the Palaeocene or Eocene (e.g. [9,11,13]) but these studies do not shed any light on when the morphological specializations seen in extant notoryctids evolved. The specimens of *N. philcreaseri* described here demonstrate that notoryctids were incipiently zalambdodont and at least semi-fossorial by the early Miocene. The early Miocene Riversleigh Faunal Zone B faunal assemblages containing *N. philcreaseri* appear to represent rainforest biotas for reasons discussed above. This led some of us (e.g. [1,33,61]) to suggest, in contrast to our earlier presumption (e.g. [22]), that notoryctids, despite being confined today to Australia's sandy deserts [53], may actually have evolved burrowing adaptations in soft rainforest floors. We suggest that as a consequence, burrowing notoryctids were serendipitously preadapted in terms of strategies for avoiding physiological stresses, to the drier environments that developed in central Australia from the late Miocene onwards as rainforests gradually retreated to coastal margins and aridity gripped Australia's heart with the first sandy deserts developing around 1 million years ago [23,24,62].
